# Insights into the role of mitophagy in lung cancer: current evidence and perspectives

**DOI:** 10.3389/fphar.2024.1420643

**Published:** 2024-06-19

**Authors:** Xin Zhang, Dongzhi Yu, Peng Tang, Fengshou Chen

**Affiliations:** ^1^ Department of Thoracic Surgery, The First Hospital of China Medical University, Shenyang, Liaoning, China; ^2^ Department of Anesthesiology, The First Hospital of China Medical University, Shenyang, Liaoning, China

**Keywords:** autophagy, mitophagy, lung cancer, tumorigenesis, treatment, progression and metastasis

## Abstract

Lung cancer, recognized globally as a leading cause of malignancy-associated morbidity and mortality, is marked by its high prevalence and lethality, garnering extensive attention within the medical community. Mitophagy is a critical cellular process that plays a crucial role in regulating metabolism and ensuring quality control within cells. Its relevance to lung cancer has garnered significant attention among researchers and scientists. Mitophagy’s involvement in lung cancer encompasses its initiation, progression, metastatic dissemination and treatment. The regulatory landscape of mitophagy is complex, involving numerous signaling proteins and pathways that may exhibit aberrant alterations or mutations within the tumor environment. In the field of treatment, the regulation of mitophagy is considered key to determining cancer chemotherapy, radiation therapy, other treatment options, and drug resistance. Contemporary investigations are directed towards harnessing mitophagy modulators, both inhibitors and activators, in therapeutic strategies, with an emphasis on achieving specificity to minimize collateral damage to healthy cellular populations. Furthermore, molecular constituents and pathways affiliated with mitophagy, serving as potential biomarkers, offer promising avenues for enhancing diagnostic accuracy, prognostic assessment, and prediction of therapeutic responses in lung cancer. Future endeavors will also involve investigating the impact of mitophagy on the composition and function of immune cells within the tumor microenvironment, aiming to enhance our understanding of how mitophagy modulates the immune response to lung cancer. This review aims to comprehensively overview recent advancements about the role of mitophagy in the tumor genesis, progenesis and metastasis, and the impact of mitophagy on the treatment of lung cancer. We also discussed the future research direction of mitophagy in the field of lung cancer.

## 1 Introduction

Lung cancer represents the foremost cause of oncology-related mortality globally, with approximately 2.1 million new cases and 1.8 million deaths each year (Garg et al., 2020). The clinical importance of lung cancer is highlighted not only by its high rates of incidence and mortality, but also by the difficulties in detecting it at an early stage. Lung cancer is mostly diagnosed in advanced stages, after significant disease progression, which greatly limits the effectiveness of current treatments. The complexities of lung cancer are further complicated by its diverse pathological classifications, with non-small cell lung cancer (NSCLC) being the most common type. (Yan et al., 2024). The treatment and prognosis of lung cancer are significantly influenced by various factors, including the patient’s genetic predisposition, molecular characteristics of the tumor, and metabolic status of the cancer cells.

In recent years, the elucidation of cellular metabolic pathways’ roles in the progression of lung cancer has garnered increasing attention. In this context, mitophagy, a quintessential process for cellular metabolic regulation and quality assurance, has been recognized as a critical mechanism (Zhou et al., 2023). This specialized autophagic process is indispensable for the removal of impaired or non-functional mitochondria, thereby safeguarding cellular energetic equilibrium and metabolic stability (Filippelli et al., 2022). Under physiological conditions, mitophagy serves as a protective mechanism, forestalling oxidative stress and cellular demise consequent to mitochondrial malfunctions (Wang et al., 2024). Mitophagy is instrumental in sustaining the cellular milieu’s stability and the equilibrium of cellular energetics through the selective eradication of impaired or non-functional mitochondria. Within the ambit of oncogenesis, mitophagy has a dual function. On one side, it contributes to carcinogenesis by promoting cellular migration, maintaining cancer stemness, and fostering resistance to pharmacological interventions. Conversely, the induction of mitophagy through specific pharmacological agents has been shown to disrupt normal cellular metabolic processes, trigger cellular stress responses, and induce genetic alterations by exacerbating mitochondrial dysfunction, ultimately leading to an antitumor effect (Qiu et al., 2021). As mitophagy is increasingly recognized as a crucial mechanism in the development and progression of lung cancer, its dysregulation within the context of lung cancer can have significant consequences. These include influencing tumor cell proliferation, viability, resistance to drugs, and metastatic capabilities. Understanding the complex role of mitophagy in lung cancer is essential for developing targeted therapeutic strategies and improving patient outcomes.

This review provides a comprehensive summary of the involvement of mitophagy in the initiation, progression, and therapeutic approaches of lung cancer, along with the underlying mechanisms elucidated by recent studies. Additionally, the challenges and future research directions of mitophagy in lung cancer are also discussed.

## 2 Overview of mitophagy

Mitophagy, a critical cellular process, involves the selective degradation of mitochondria through autophagy, and plays a crucial role in maintaining mitochondrial quality control and cellular homeostasis (Pickles et al., 2018; Filippelli et al., 2022; Yang et al., 2024).

Mitophagy is initiated by a complex signaling pathway in response to various cellular stresses, such as mitochondrial damage, oxidative stress, or energy depletion (Arora et al., 2022). The primary modalities that govern mitophagy encompass ubiquitin-dependent, receptor dependent, and other pathways (Ma et al., 2023; Tang et al., 2024). The process begins with the recognition and tagging of damaged or dysfunctional mitochondria by specific proteins, such as Parkin, proteins like PTEN-induced kinase 1 (PINK1), Bcl-2/adenovirus E1B 19-kDa-interacting protein 3 (BNIP3), and Nix (Wu et al., 2023; Yang et al., 2024). These proteins work together to target the damaged mitochondria for degradation. Once tagged, the damaged mitochondria are engulfed by a double-membraned structure called the autophagosome (Lu et al., 2023). This structure then fuses with a lysosome, forming an autolysosome (Lu et al., 2023). The lysosome contains enzymatic machinery that degrades the contents of the autophagosome, including the damaged mitochondria. Mitophagy eliminates dysfunctional mitochondria, which could otherwise lead to the accumulation of toxic molecules and the induction of cellular apoptosis (Chourasia et al., 2015). It also allows for the recycling of damaged mitochondria components, such as proteins and lipids, thus promoting mitochondrial renewal and maintaining cellular energy metabolism (Morán et al., 2014).

## 3 Role of mitophagy in the development and progression of lung cancer

**FIGURE 1:**
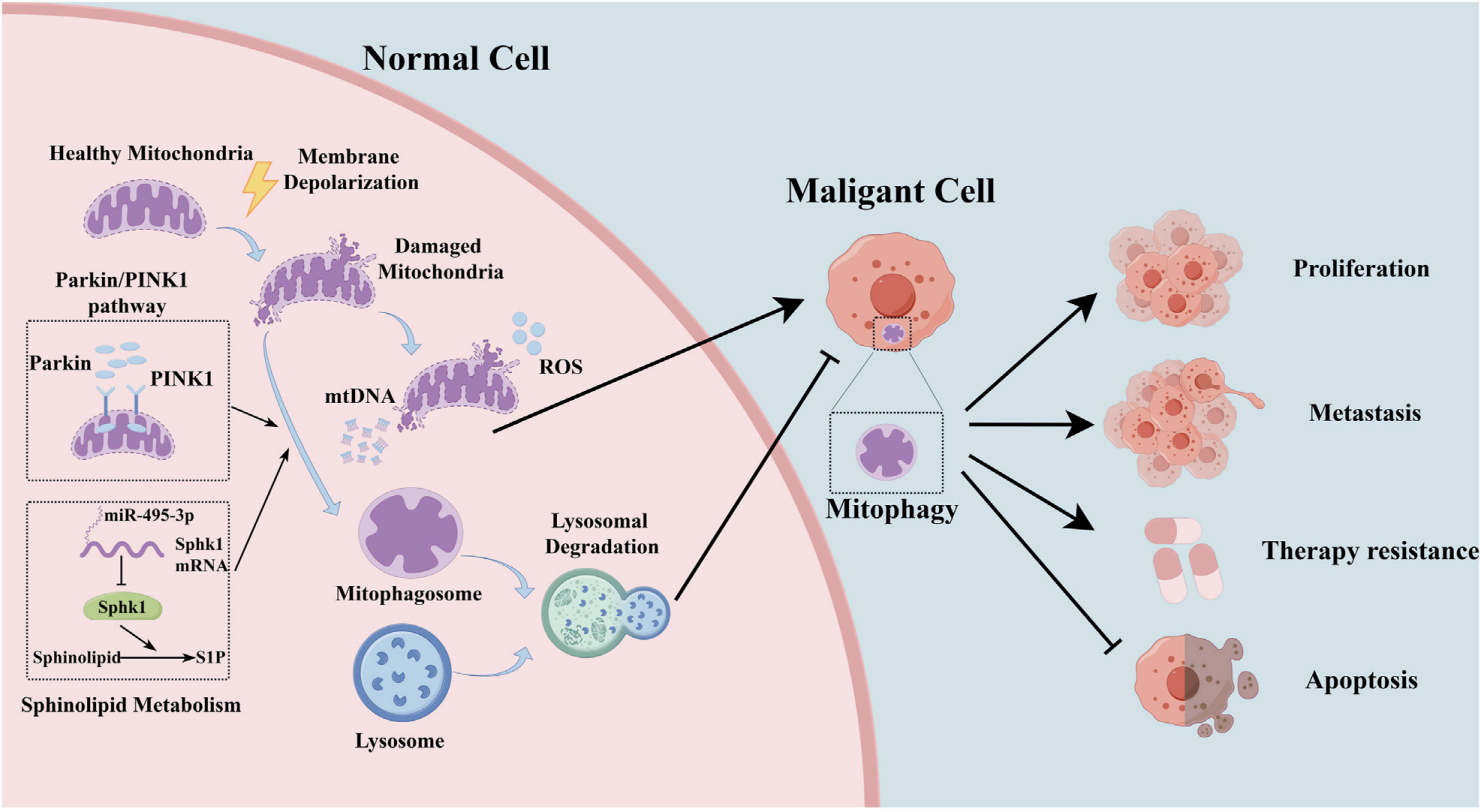
The representative mechanism of mitophagy in lung cancer.

At the molecular level, the regulatory landscape of mitophagy in lung cancer is governed by a complex network of key molecules and signaling pathways. (Berthier et al., 2011; Liu M. et al., 2021; Arora et al., 2022). Within the milieu of lung cancer, disruptions or dysfunctions in these signaling pathways can significantly alter the accuracy and efficiency of mitophagy. These regulatory perturbations can profoundly impact tumor cell behavior and the course of disease progression, highlighting the complex interaction between cellular homeostasis mechanisms and oncogenic processes (Figure 1; Table 1).

**TABLE 1:** Role of mitophagy in lung cancer.

Potential targets	Tissues/Cells	Signaling	Function	References
Parkin	Human lung adenocarcinomas/A549 cell line	-	Different parkin isoforms are expressed in human lung adenocarcinomas. Some of them are also present in A549 cell line	D’Amico et al. (2015)
Pink1	Squamous carcinoma of lung	-	Diffuse cytoplasmic expression of Pink1 in SQCC contrasts with the granular cytoplasmic pattern in normal lung tissues	Berthier et al. (2011)
Sphk1	A549, H1299 cell line	miR-495–3p/Sphk1	Induces lethal mitophagy to suppressNSCLC tumorigenesis	Arora et al. (2022)
TP53	NSCLC Tumor tissues	-	TP53-p.Glu358Val as a driver mutation that activates mitophagy to support cancer cell growth	Wang et al. (2022)
CAV1, DSG2, DSP, MYH11, NME1, PAICS, PLOD2 (Seven Mitophagy and aging-related genes)	Lung adenocarcinoma	-	Mitophagy and aging-related genes linked with the prognosis of lung adenocarcinoma patients	Meng et al. (2023)
FIS1	Lung cancer stem cells	-	Enhancement of mitophagy in lung CSCs, induced by FIS1 through mitochondrial fission, correlates with diminished overall survival	Liu et al. (2021a)
TLR9	Lung cancer stem cells	Notch1/AMPK	Lung cancer stem-like cells show high levels of mitophagy, resulting in lysosomal mtDNA accumulation that activates TLR9 and Notch1-AMPK signaling. The TLR9-Notch1-AMPK pathway promotes CSC expansion and can be therapeutically targeted	Liu et al. (2023b)

SQCC, lung squamous cell carcinoma; NSCLC, non-small cell lung cancer; CAV1, caveolin 1; DSG2, desmoglein 2; DSP, desmoplakin; MYH11, myosin heavy chain 11; NME1, NME/NM23 nucleoside diphosphate kinase 1; PAICS, phosphoribosylaminoimidazole carboxylase and phosphoribosylaminoimidazolesuccinocarboxamide synthetase; PLOD2, procollagen-lysine two-oxoglutarate; FIS1, fission 1.

### 3.1 The tumorigenesis of lung cancer and mitophagy

In early-stage lung cancer, there are significant alterations in the cell environment, including augmented oxidative stress and disturbances in energy metabolism (LeBleu et al., 2014). Mitophagy plays a critical role in maintaining mitochondrial balance by removing damaged mitochondria (Chen et al., 2024). As a result, activating mitophagy helps eliminate damaged mitochondria, reducing the release of reactive oxygen species (ROS). This process is crucial for maintaining energy balance and reducing oxidative stress, which could potentially prevent the oncogenic transformation of cells (Song et al., 2023). Impaired mitophagy leads to decreased elimination of damaged mitochondria, resulting in elevated ROS production and the buildup of mitochondrial DNA in the cytoplasm (Ng Kee Kwong et al., 2017). Mitophagy may play a tumor-suppressive role by reducing excessive ROS production and inhibiting inflammasome activation (Morselli et al., 2011; Ng Kee Kwong et al., 2017). Parkin, an essential E3 ubiquitin ligase, is activated by phosphorylated ubiquitin and plays a crucial role in orchestrating the polyubiquitination of a wide array of substrates (Morselli et al., 2011; McWilliams and Muqit, 2017). Proteins like PTEN-induced kinase 1 (PINK1) and Parkin function as key regulators in detecting mitochondrial stress and initiating autophagic responses (Berthier et al., 2011). Disruption in the Pink1/Parkin mitophagy pathway, also observed in lung cancer, plays a role in the pathogenesis of chronic obstructive pulmonary disease (COPD) (Mizumura et al., 2014; D’Amico et al., 2015). The diminution of Parkin in COPD-afflicted lungs correlates with increased ROS and senescence in bronchial epithelial cells (Ito et al., 2015). Notably, diffuse cytoplasmic expression of Pink1 in lung squamous cell carcinoma (SQCC) contrasts with the granular cytoplasmic pattern in normal lung tissues, implicating aberrant Pink1 expression in lung carcinogenesis (Berthier et al., 2011). Human lung adenocarcinomas exhibit variable Parkin isoforms, potentially modulating apoptosis, mitophagy, and mitochondrial fusion (D'Amico et al., 2015). Sphingolipid metabolites, specifically ceramide and sphingosine-1-phosphate (S1P), play a crucial role in cellular proliferation and apoptosis. Sphk1, a key enzyme converting sphingosine into S1P, promotes cell proliferation and survival (Ogretmen, 2018). MiR-495–3p, by targeting Sphk1, shifts the sphingolipid balance towards ceramide, inducing lethal mitophagy to inhibit NSCLC tumorigenesis (Arora et al., 2022). These studies indicate that mitophagy has a dual role in the early stages of lung cancer development, serving as both a protective mechanism and a facilitator of cancer initiation (Gozuacik and Kimchi, 2004).

### 3.2 Mitophagy in lung cancer progression and metastasis

As lung cancer advances, the intricacies of mitophagy’s role become increasingly complex. Mitochondria, pivotal for intracellular energy metabolism, assume a critical role, especially in tumor cells, given their elevated energy requisites (Liu J. et al., 2023). This process meticulously governs cellular metabolic states and energy generation, profoundly influencing the proliferation and division of lung cancer cells (Zhu et al., 2022). Enhanced mitophagy furnishes additional energy requisite for the expedited proliferation and growth of cancer cells (Qiu et al., 2021). Simultaneously, it occupies a central role in cellular death mechanisms, encompassing apoptosis and necrosis (Li et al., 2023). Under certain conditions, lung cancer cells may invoke mitophagy to evade apoptosis, engendering heightened drug resistance and survival (Liu D. et al., 2021; Liu Z. et al., 2023). Moreover, the metastatic process in cancer is intricately linked with mitophagy (He et al., 2023). Successful metastasis necessitates not only ample energy for cancer cells but also alterations in cell-to-cell interactions and migratory capabilities. Mitophagy modulates these aspects by influencing various intracellular signaling pathways and molecules, thereby affecting cellular adhesion, motility, and invasive potential (Liu D. et al., 2021; Wu et al., 2022; Liu Z. et al., 2023; Li et al., 2023).

The TP53 gene, prevalently mutated in cancer and recognized as a tumor suppressor, is mutated in half of NSCLC cases (Mogi and Kuwano, 2011; Nguele Meke et al., 2024). A next-generation sequencing (NGS) study on tumor tissues from 314 Chinese NSCLC patients delineated the mutational landscape in NSCLC, identifying TP53-p.Glu358Val as a driver mutation that activates mitophagy to support cancer cell growth (Wang et al., 2022). Pharmacological inhibition of autophagy/mitophagy selectively curtails the proliferation of TP53-null or TP53-p.Glu358Val-expressing lung cancer cells (Wang et al., 2022). Mitophagy and aging (MiAg)-related genes are pivotal in tumors and prognostication for various cancer types. Seven MiAg-related genes—caveolin 1(CAV1), desmoglein 2 (DSG2), desmoplakin (DSP), myosin heavy chain 11 (MYH11), NME/NM23 nucleoside diphosphate kinase 1 (NME1), phosphoribosylaminoimidazole carboxylase and phosphoribosylaminoimidazolesuccinocarboxamide synthetase (PAICS), procollagen-lysine 2-oxoglutarate 5-dioxygenase 2(PLOD2)—have been significantly linked with the prognosis of lung adenocarcinoma (LUAD) patients (Meng et al., 2023). Cancer stem cells (CSCs), first identified in the hematopoietic system and subsequently in various solid tumors, exhibit self-renewal and differentiation capacities, significantly influencing tumorigenesis, metastasis, and recurrence (Bao et al., 2013; Liu D. et al., 2021). Mitochondria, serving as the energy reservoir for cells, and mitophagy, are vital for CSC survival (Lee et al., 2018). Enhancement of mitophagy in lung CSCs, induced by fission-1 (FIS1) through mitochondrial fission, correlates with diminished overall survival (Liu D. et al., 2021). Hypermitophagy characterizes human lung CSCs, fostering metabolic adaptation via the Notch1-AMPK axis to propel CSC expansion (Liu Z. et al., 2023).

## 4 The relationship between mitophagy and lung cancer treatment

### 4.1 The growing significance of mitophagy in lung cancer therapeutics

The nuanced role of mitophagy in lung cancer therapeutics has emerged as a focal point in modern oncological discourse. Its critical influence on the biological dynamics of lung cancer cells, significantly impacting the efficacy of diverse therapeutic approaches, is increasingly acknowledged (Table 2).

**TABLE 2:** The relationship between mitophagy and lung cancer treatment.

Cells	Signaling	Relationship between mitophagy and lung cancer treatment	References
HCC827, A540, and H1299	PINK1/Parkin	Erastin and celastrol instigates ATG5/ATG7-dependent autophagy, PINK1/Parkin-mediated mitophagy, and the induction of HSPs in an HSF1-dependent manner. The suppression of HSF1 further intensifies cell death in NSCLC cell lines HCC827, A540, and H1299, and impedes tumor proliferation *in vivo*	Liu et al. (2021b)
A549 and H1299 cells	-	PHB2 reduced parkin-mediated mitophagy, which suppressed proliferation and migration of A549 and H1299 cells	Zhang et al. (2020a)
A549 cell line	-	DFP, an iron chelator that can induce mitophagy, greatly increased the death of A46T Parkin-expressing lung cancer cells	Zhang et al. (2020b)
A549 cell line		DHE, known for its antimigraine properties, triggers lung cancer cell demise through apoptosis and mitophagy induction	Chang et al. (2016)
A549 cell line		Ursolic and oleanolic acids, widespread in plants and fruits, exhibit anticancer properties and induce mitophagy in A549 human lung cancer cells	Castrejón-Jiménez et al. (2019)
A549 cell line		Depletion of PINK1 in A549 cells via shRNA reduces cell proliferation, augments cell death, diminishes ATP production, inhibits mitophagy, and increases ROS alongside caspase-9-dependent apoptosis	Dai et al. (2019)
A549 cell line		Mitophagy was found to be induced by resveratrol and mitophagy was mediated by LC3B/p62 interaction and could be inhibited by LC3B knockout and p62 knockdown following increased apoptosis in A549 cells	Zheng et al. (2021)
A549 cell line		APE1 promotes the cisplatin resistance of lung cancer cells by inducing Parkin-mediated mitophagy	Li et al. (2019)
A549 cell line		CAV1 silencing augments cisplatin sensitivity via inhibition of Parkin-related mitophagy and activation of the ROCK1 pathway	Liu et al. (2020)
H69, H69AR (a cell line induced by H69 with doxorubicin), H446, and HBE cell line	METTL3/DAP2/Pink1/Parkin	The m6A methyltransferase METTL3 regulates Pink1-Parkin pathway-mediated mitophagy and mitochondrial damage in SCLC cells by targeting DCP2, thereby promoting chemotherapy resistance in patients with SCLC.	Sun et al. (2023)
HEK293FT cell line	-	BEX2 expression is elevated after anticancer drug treatment, and its overexpression inhibits chemotherapy-induced apoptosis. In addition, inhibition of BEX2-regulated mitophagy sensitizes tumor cells to apoptosis	Mu et al. (2023)
H1975 and PC9 cell line		The circular RNA IGF1R (cIGF1R), encoded by IGF1R, serves as a molecular switch that limits the mitophagy of drug-tolerant persister tumor cells in NSCLC	Wang et al. (2023)
A549, SPC-A1, NCI-H460 and NCI-H520 cell line		Temozolomide-perillyl alcohol conjugate (TMZ-POH) disrupts mitophagy flux by eliciting lysosomal dysfunction within NSCLC cells, thereby augmenting their sensitivity to radiation therapy	Chang et al. (2018)
A549 cell line	CIRBP/PINK1/Parkin	DHA reduces radiation-induced mitophagy and radioresistance of lung cancer A549 cells via CIRBP/PINK1/Parkin pathway	Wu et al. (2022)

ATG5/ATG7, autophagy-related 5/autophagy-related 7; HSPs, heat shock proteins; NSCLC, non-small cell lung cancer; DFP, deferiprone; APE1, apurinic/apyrimidinic endonuclease 1; CAV1, caveolin 1; ROCK1, rho-associated coiled-coil kinases 1; METTL3, methyltransferase-like 3; DAP2, dipeptidyl aminopeptidase; DCP2, decapping mRNA, 2; SCLC, small-cell lung cancer; BEX2, brain expressed X-linked 2; TMZ-POH, temozolomide-perillyl alcohol conjugate; DHA, dihydroartemisinin; CIRBP, cold inducible RNA, binding protein.

Melittin, known for its robust surface activity on lipid membranes, interacts with membranes and induces membrane fragmentation (Li et al., 2023). Recently, melittin has demonstrated promising therapeutic effects in various tumors, including glioblastoma (Ertilav and Nazıroğlu, 2023), breast cancer (Daniluk et al., 2023), and melanoma (Jeong et al., 2023). It targets mitochondria and impedes mitophagy flux in adenocarcinoma alveolar cancer (A549) cell lines (Li et al., 2023). Erastin, a classical inducer of ferroptosis, exhibits promising pharmacological effects in cancer therapeutics (Mou et al., 2019). Celastrol, derived from the traditional Chinese medicinal herb Tripterygium wilfordii, known as Thunder God Vine, has demonstrated potent antitumor activities across various cancer cell lines and *in vivo* models (Tang et al., 2015). The synergistic application of erastin with celastrol precipitates cell death at subtoxic concentrations, amplifying reactive oxygen species (ROS) production, perturbing mitochondrial membrane potential, and facilitating mitochondrial fission. Moreover, the concurrent administration of erastin and celastrol instigates autophagy-related 5 (ATG5)/ATG7-dependent autophagy, PINK1/Parkin-mediated mitophagy, and the induction of heat shock proteins (HSPs) in a heat shock factor 1 (HSF1)-dependent manner (Liu M. et al., 2021). The suppression of HSF1 further intensifies cell death in NSCLC cell lines HCC827, A540, and H1299, and impedes tumor proliferation *in vivo* (Liu M. et al., 2021). Prohibitin 2 (PHB2), situated in the inner mitochondrial membrane (IMM), functions as a mitophagy receptor (Wei et al., 2017). Elevated PHB2 levels in human NSCLC specimens, relative to adjacent non-tumor tissues, have been observed. The inhibition of PHB2 expression attenuates mitophagy in A549 and human lung adenocarcinoma (H1299) cells, as evidenced by reduced LC3 II/I and parkin markers and increased p62 protein levels. The downregulation of PHB2 diminishes parkin-mediated mitophagy, curbing the proliferation and migration of A549 and H1299 cells (Zhang H. et al., 2020). Typically, Parkin is dispersed throughout the nucleus and cytosol, relocating to damaged mitochondria under stress to facilitate the ubiquitination of mitochondrial proteins and instigate mitophagy (Harper et al., 2018). Mutations in the tumor suppressor gene PARK2 disrupt PINK1/Parkin-mediated mitophagy in lung cancer cells and deferiprone (DFP), an iron chelator that can induce mitophagy, greatly increased the death of A46T Parkin-expressing lung cancer cells (Zhang Z. L. et al., 2020). Dihydroergotamine tartrate (DHE), known for its antimigraine properties, triggers lung cancer cell demise through apoptosis and mitophagy induction (Chang et al., 2016). Ursolic and oleanolic acids, widespread in plants and fruits, exhibit anticancer properties and induce mitophagy in A549 human lung cancer cells (Castrejón-Jiménez et al., 2019). The depletion of PINK1 in A549 cells via shRNA reduces cell proliferation, augments cell death, diminishes ATP production, inhibits mitophagy, and increases ROS alongside caspase-9-dependent apoptosis (Dai et al., 2019). Cells deficient in PINK1 exhibit heightened sensitivity to the glycolytic inhibitor 3-bromopyruvate (3-BP), further disrupting ATP synthesis (Dai et al., 2019). Resveratrol (Res), a polyphenol phytoalexin, is recognized for its antitumorigenic and chemopreventive properties (Jang et al., 1997). Res induces non-canonical autophagy and apoptosis in A549 lung cancer cells, whereas LC3B/p62-mediated mitophagy shields tumor cells from apoptosis, elucidating the pivotal role of mitophagy in determining cell fate (Zheng et al., 2021).

### 4.2 Mitophagy and chemotherapy

Chemotherapy remains a fundamental strategy in lung cancer management, yet it frequently confronts the obstacle of tumor cell resistance to pharmacological interventions (Gridelli et al., 2005; Bao et al., 2013). In this milieu, mitophagy assumes a complex role, influencing the efficacy of chemotherapeutic regimens (Gridelli et al., 2005; Li et al., 2019; Liu et al., 2020). Cisplatin, a quintessential chemotherapeutic agent, is widely administered across a spectrum of solid tumors, encompassing testicular, head and neck, ovarian, esophageal, cervical, and non-small cell lung cancer (NSCLC) (Kelland, 2007). The pervasiveness of cisplatin resistance, however, compromises the therapeutic success in advanced NSCLC treatments (Gridelli et al., 2005). Apurinic/apyrimidinic endonuclease 1 (APE1), a pivotal multi-functional DNA repair enzyme, is integral for DNA damage repair, redox regulation, and transcription factor activity modulation (Li and Wilson, 2014). The mitochondrial translocation of APE1 enhances the mitochondrial membrane potential, diminishes cytochrome c levels, and triggers Parkin-mediated mitophagy, contributing to cisplatin resistance in lung cancer cells (Li et al., 2019). Conversely, Caveolin-1 (Cav-1) expression is significantly reduced in A549 lung cancer cells following cisplatin exposure, where Cav-1 silencing augments cisplatin sensitivity via inhibition of Parkin-related mitophagy and activation of the Rho-associated coiled-coil kinases 1 (ROCK1) pathway (Liu et al., 2020). Parkin-independent mitophagy also dictates the chemotherapeutic response in various cancers, notably breast and lung adenocarcinomas (Villa et al., 2017). While small cell lung cancer (SCLC) patients initially respond to platinum-based chemotherapy, durable responses are rare, frequently leading to chemoresistance and disease recurrence (Bao et al., 2013). Methyltransferase-like 3 (METTL3), a prominent m6A methyltransferase, influences a myriad of biological processes, including proliferation and migration (Zaccara et al., 2019). It modulates the Pink1-Parkin pathway-mediated mitophagy and mitochondrial damage in SCLC cells by targeting decapping mRNA 2 (DCP2), thereby facilitating chemoresistance in SCLC patients (Sun et al., 2023). Additionally, the BEX2 gene, part of the brain-expressed X-linked gene family, through crotonylation, interacts with NDP52 to augment mitophagy, influencing chemotherapeutic-induced apoptosis in NSCLC cells (Naderi, 2019; Mu et al., 2023). The circular RNA IGF1R (cIGF1R), encoded by IGF1R, serves as a molecular switch that limits the mitophagy of drug-tolerant persister tumor cells in NSCLC (Wang et al., 2023). Modulating mitophagy offers a promising avenue to enhance lung cancer cell sensitivity to chemotherapeutic agents, potentially circumventing the perennial challenge of drug resistance.

### 4.3 Radiotherapy and mitophagy

Mitophagy is garnering growing attention in lung cancer radiotherapy. Radiation therapy provokes DNA damage and oxidative stress within cellular structures, and mitophagy plays a pivotal role in mitigating these adverse effects (Chang et al., 2018; Wu et al., 2022). The Temozolomide-perillyl alcohol conjugate (TMZ-POH), an innovative derivative synthesized through the amalgamation of Temozolomide (TMZ) and perillyl alcohol (POH), has demonstrated pronounced anticancer efficacy across a spectrum of malignancies (Cho et al., 2012). TMZ-POH disrupts mitophagy flux by eliciting lysosomal dysfunction within Non-Small Cell Lung Cancer (NSCLC) cells, thereby augmenting their sensitivity to radiation therapy (Chang et al., 2018). Dihydroartemisinin (DHA), recognized for its anticancer properties and minimal toxicity, is increasingly being explored in both preclinical and clinical settings as an anticancer agent or a therapeutic adjuvant (Li et al., 2021; Wu et al., 2022). DHA attenuates radiation-induced mitophagy and radioresistance in lung cancer A549 cells through the inhibition of the Cold-Inducible RNA Binding Protein (CIRBP), offering new avenues for enhancing the effectiveness of radiotherapy in lung cancer treatment (Wu et al., 2022).

## 5 Challenges and future research directions in mitophagy for lung cancer

Mitophagy, an area of pivotal significance in lung cancer research, has witnessed notable advancements yet faces myriad challenges and uncharted territories. These challenges encompass the complexity of mitophagy mechanisms, its incorporation into lung cancer diagnostics, therapeutic strategies, and prognostic assessments.

### 5.1 Challenges in mitophagy for lung cancer

Mitophagy holds promise for lung cancer therapy due to its role in cellular homeostasis and apoptosis. However, several challenges must be addressed to harness its potential:

1. The intricate regulatory mechanisms of mitophagy in lung cancer are not fully understood, complicating the development of targeted interventions.
2. Mitophagy can either suppress or promote tumor growth depending on the context, making it challenging to predict its therapeutic impact.
3. Establishing a direct link between mitophagy activity and lung cancer patient outcomes is difficult due to the disease’s heterogeneity and the challenges in measuring mitophagy in clinical settings.
4. Creating drugs that selectively modulate mitophagy in lung cancer cells without affecting healthy cells is a significant challenge.
5. Understanding and overcoming the role of mitophagy in drug resistance is crucial for improving treatment efficacy.
6. The interaction between mitophagy and immune cell function within the tumor microenvironment is complex. The challenge lies in leveraging this interaction to enhance the effectiveness of immunotherapies.
7. Bridging the gap between preclinical findings and clinical application involves overcoming significant translational research barriers, including safety, efficacy, and regulatory hurdles.
8. The diverse genetic and environmental backgrounds of lung cancer patients complicate the development of personalized mitophagy-based therapies.

### 5.2 Future research directions in mitophagy for lung cancer

Although current studies enhance our understanding of the role of mitochondrial homeostasis in lung cancer, there are still some key questions about the process and function of mitophagy in lung cancer.

1. Further research is needed to dissect the molecular pathways that regulate mitophagy in lung cancer cells. Understanding these mechanisms could reveal novel therapeutic targets.
2. Studies should explore how mitophagy influences the composition and function of the immune cells within the tumor microenvironment. This could provide insights into how mitophagy modulates the immune response against lung cancer.
3. The development and testing of mitophagy modulators, either as standalone treatments or in combination with existing therapies, could enhance the efficacy of lung cancer treatments.
4. Research should focus on how mitophagy contributes to the development of drug resistance in lung cancer, potentially leading to strategies to overcome this barrier.
5. Identifying biomarkers related to mitophagy could improve early detection and prognosis of lung cancer, potentially leading to more personalized treatment approaches.
6. Investigating the relationship between mitophagy and metastasis could uncover new strategies to prevent or treat the spread of lung cancer.

## 6 Conclusion

Mitophagy has ascended to prominence within the domain of lung cancer research, serving as a pivotal mechanism for maintaining cellular energy equilibrium and metabolic integrity. It accomplishes this by targeting and removing damaged or dysfunctional mitochondria, thereby exerting a profound influence on the growth, survival, and chemoresistance of lung cancer cells. The role of mitophagy is integral to the initiation, advancement, and dissemination of lung cancer, with its regulatory network comprising an array of signaling molecules and pathways. These components are subject to potential dysregulation or mutations within the context of lung cancer, further complicating the disease’s pathology. Further exploration of these issues may facilitate the development of novel strategies for lung cancer prevention and treatment.
